# Sirtuin 3 Restores Synthesis and Secretion of Very Low-Density Lipoproteins in Cow Hepatocytes Challenged with Nonesterified Fatty Acids In Vitro

**DOI:** 10.3390/vetsci8070121

**Published:** 2021-06-30

**Authors:** Dongmei Xing, Baogen Wang, Hong Lu, Tao Peng, Jianming Su, Hongyu Lei, Jianhua He, Yingfang Zhou, Lei Liu

**Affiliations:** 1 Hunan Provincial Key Laboratory of Protein Engineering in Animal Vaccines, College of Veterinary Medicine, Hunan Agricultural University, Changsha 410128, China; salrene@163.com (D.X.); wbg02468@stu.hunau.edu.cn (B.W.); red19950802@163.com (H.L.); lwp@hunau.net (T.P.); sjmauhn@hunau.edu.cn (J.S.); leihy77@hunau.edu.cn (H.L.); 2 College of Animal Science and Technology, Hunan Agricultural University, Changsha 410128, China; jianhuahy@hunau.edu.cn

**Keywords:** animal metabolic diseases, fat metabolism, MTP, ApoB100, ApoE, perinatal period

## Abstract

Fatty liver is closely associated with elevated concentrations of nonesterified fatty acids (NEFA) and a low level of very low-density lipoproteins (VLDL) in blood of dairy cows. High NEFA inhibit the VLDL synthesis and assembly, and cause hepatic triacylglycerol (TAG) deposition. Sirtuin 3 (SIRT3), a mitochondrial deacetylase, antagonizes NEFA-induced TAG accumulation through modulating expressions of fatty acid synthesis and oxidation genes in cow hepatocytes. However, the role of SIRT3 in the VLDL synthesis and assembly was largely unknown. Here we aimed to test whether SIRT3 would recover the synthesis and assembly of VLDL in cow hepatocytes induced by high NEFA. Primary cow hepatocytes were isolated from 3 Holstein cows. Hepatocytes were infected with SIRT3 overexpression adenovirus (Ad-SIRT3), SIRT3-short interfering (si) RNA, or first infected with Ad-SIRT3 and then incubated with 1.0 m*M* NEFA (Ad-SIRT3 + NEFA). Expressions of key genes in VLDL synthesis and the VLDL contents in cell culture supernatants were measured. SIRT3 overexpression significantly increased the mRNA abundance of microsomal triglyceride transfer protein (MTP), apolipoprotein B100 (ApoB100) and ApoE (*p <* 0.01), and raised VLDL contents in the supernatants (*p* < 0.01). However, SIRT3 silencing displayed a reverse effect in comparison to SIRT3 overexpression. Compared with NEFA treatment alone, the Ad-SIRT3 + NEFA significantly upregulated the mRNA abundance of MTP, ApoB100 and ApoE (*p* < 0.01), and increased VLDL contents in the supernatants (*p* < 0.01). Our data demonstrated that SIRT3 restored the synthesis and assembly of VLDL in cow hepatocytes challenged with NEFA, providing an in vitro basis for further investigations testing its feasibility against hepatic TAG accumulation in dairy cows during the perinatal period.

## 1. Introduction

Fatty liver is one of the most destructive metabolic diseases in dairy cows [1]. It is defined by the excessive accumulation of triacylglycerol (TAG) in the liver, and is related to declined health status, productivity, and reproductive performance [2]. Fatty liver can be further classified into three categories based on the liver TAG levels, i.e., mild fatty liver (1–5% liver TAG, % wet weight), moderate fatty liver (5–10% liver TAG), and severe fatty liver (>10% liver TAG) [3]. The incidence of fatty liver is high globally, ranging from 20% to 65% for moderate fatty liver and 5% to 20% for severe fatty liver [3]. The mortality rate of fatty liver cases is high because of lack of effective therapy [4].

Fatty liver takes place once the rate of hepatic TAG production exceeds the rate of TAG clearance [5]. In terms of TAG clearance, the synthesis and assembly of very low-density lipoproteins (VLDL) play a pivotal role in maintaining lipid homeostasis in bovine hepatocytes. There are several important proteins involved in the synthesis and assembly of VLDL. These proteins include but are not limit to microsomal triglyceride transfer protein (MTP), apolipoprotein B100 (ApoB100), and ApoE [2,5]. Through VLDL, bovine hepatocytes transport endogenous lipids to extrahepatic tissues and therefore prevent cellular TAG deposition. Unfortunately, compared to rodents, ruminants possess a low rate of hepatic VLDL secretion [6,7], making them vulnerable to fat deposition. Moreover, cows with fatty liver display lower contents of TAG-rich lipoprotein in blood [8,9]. We previously revealed that high NEFA suppressed the synthesis and assembly of VLDL and induced TAG accumulation in primary cow hepatocytes [10], supporting that high concentration of blood NEFA was a pathologic basis for the development of fatty liver. Those observations highlight a critical piece of limited VLDL synthesis and assembly in the development of fatty liver during the perinatal period.

Sirtuins 3 (SIRT3), a type of nicotinamide adenine dinucleotide (NAD^+^)-dependent deacetylases, regulates cellular energy metabolism and redox homeostasis in nonruminants [11]. Hepatic SIRT3 expression decreased in the first two weeks after calving in dairy goats [12]; hepatic SIRT3 content was lower in cows with fatty liver compared to healthy controls [13]. Additionally, overexpression of SIRT3 alleviates TAG deposition induced by high NEFA via inhibition of fatty acid synthesis and promotion of fatty acid oxidation in cow hepatocytes [13]. Accumulating evidence shows that SIRT3 may serve as an antagonist against the pathophysiological events induced by high NEFA [12,13]. Thus far, the role of SIRT3 in the VLDL synthesis and assembly has not been investigated extensively. Here we aimed to study SIRT3’s effect on the VLDL synthesis and assembly of cow hepatocytes in response to high NEFA.

## 2. Material and Methods

### 2.1. Construction of Recombinant Adenoviruses and siRNA

The empty adenovirus vector (Ad-green fluorescent protein, Ad-GFP), SIRT3 overexpression adenovirus (Ad-SIRT3), and short interfering RNA (siRNA) were constructed by Hanbio (Shanghai, China) as described elsewhere [13]. Briefly, the cDNA sequence of bovine SIRT3 was synthesized and further cloned into pHBAd-EF1a-MCS-3flag-CMV-EGFP vectors. The sequence of GFP was also cloned to the same vectors as negative controls. In order to get a stable SIRT3 silencing, we used an equivalent mixture of three SIRT3 siRNA sequences; siRNA sequences are shown in Table S1. The titer of adenovirus before treatment was 2 × 10^10^ pfu/mL.

### 2.2. Cow Primary Hepatocytes Culture and Treatment

We followed a similar protocol for the isolation and culture of cow hepatocytes as in our previous study [13]. The protocol was approved by the Institutional Animal Care and Use Committee of Hunan Agricultural University (Changsha, China). Cow primary hepatocytes were isolated using a three-step perfusion method. The composition of perfusion solution A, B, and C can be found elsewhere [13]. Three one-day-old Holstein females were bought from a dairy farm. Following health examination by veterinaries birth, healthy fasting animals were transported to the research facility. The caudate lobe of the liver was surgically removed, as previously described [14]. Cows were subject to general anesthesia with thiamylal sodium. A 25 cm right abdominal incision was made from the costal margin. The caudate lobe of the liver was then excised, and placed into a sterile dish. It was perfused through vessels with perfusion solution A at a flow rate of 50 mL/min for 12 min, and then by perfusion solution B at the same flow rate for 3 min. After the flow became clear, perfusion solution C containing collagenase type IV at 0.2% (*wt*/*v*) was introduced at a flow rate of 20 mL/min for about 20 min. The collagenase digestion was stopped by 100 mL of fetal bovine serum (FBS) (Hyclone Laboratories, Logan, UT, USA). After removal of the liver capsule, blood vessels, fats, and connective tissues, the remainder of liver parenchyma was minced and filtered successively through a 150 μm sieve and a 75 μm sieve. Cells were then washed twice with a RPMI-1640 basic medium (Hyclone Laboratories, Logan, UT, USA) at 4 °C, and were reconstituted in an adherent medium (basic medium with 10% FBS, 10^−6^ M insulin, 10^−6^ M dexamethasone, 10 μg/mL vitamin C), and seeded into a six-well tissue culture plate at 1 × 10^6^ cells/mL for incubation (37 °C, 5% CO_2_). After 24 h, hepatocytes were cultured in a growth medium (RPMI-1640 basic medium + 10% FBS) instead. After 48 h cultivation, hepatocytes were maintained in the basic medium for 6 h before treatments. Cells after treatments were harvested and stored at −80 °C for further use within 2 weeks.

Cow hepatocytes were infected with Ad-SIRT3/Ad-GFP at different multiplicity of infection (MOI) ratios (MOI = 0, 25, 50, 100) for 6 h and then incubated with the growth medium for another 42 h before the hepatocytes were collected. Hence, there were five groups in the present experiment: Ad-GFP (MOI = 100, negative control), Ad-SIRT3 MOI 0 (blank control), Ad-SIRT3 MOI 25, Ad-SIRT3 MOI 50, and Ad-SIRT3 MOI 100.

Three groups were included in the si-SIRT3 experiment: negative control (NC), SIRT3-siRNA (si-SIRT3), and blank control (C). The SIRT3-siRNA (an equivalent mixture of three SIRT3 siRNA sequences) and NC (negative control) -siRNA were delivered into hepatocytes according to the manual for Lipofectamine 2000 Transfection Reagent (Invitrogen, Waltham, MA, USA). The siRNA treatments lasted for 6 h. Then cells were renewed for the growth medium and incubated for another 42 h before harvest.

To investigate the effects of SIRT3 overexpression plus NEFA treatment on VLDL synthesis and assembly, cells were divided into four groups: Ad-SIRT3 + NEFA, Ad-GFP, NEFA, and blank controls. Cells in the Ad-SIRT3 + NEFA group were transfected with 100 MOI Ad-SIRT3 for 30 h, then serum starved for 6 h, and finally were coincubated with 1 m*M* NEFA in the basic medium containing 2% BSA (*w*/*v*) for 12 h. The composition of NEFA preparations was described in detail elsewhere [10]. Fatty acids were dissolved in KOH (0.1 *M*) by heating and adjusted to pH 7.4 with HCl (1 *M*). All fatty acid standards were products of Sigma-Aldrich (St. Louis, MO, USA). The stock NEFA was a mixture of C16:1 (2.8 m*M*), C18:1 (22.9 m*M*), C18:2n-6 (2.6 m*M*), C16:0 (16.8 m*M*), and C18:0 (7.6 m*M*). The negative controls (Ad-GFP group) were the hepatocytes treated with Ad-GFP (100 MOI) but without NEFA treatment in parallel with the Ad-SIRT3 + 1 m*M* NEFA group. The blank controls were the hepatocytes without any adenovirus infection and NEFA treatment. The variables introduced to cells were only the different treatments.

### 2.3. Quantitative Reverse-Transcription PCR (qRT-PCR) Assay

We followed the MIQE (Minimum Information for Publication of Quantitative Real-Time PCR Experiments) guidelines to conduct the qRT-PCR assays [15]. Cells were immediately subject to total RNA extraction after collection. Total RNA extraction was completed with RNAiso Plus reagent (D9108A, TaKaRa, Dalian, China) by following its manual. About 2 μg total RNA (after quality check) was reverse-transcribed to cDNA by a Reverse Transcription Kit (RR047A, TaKaRa, Tokyo, Japan) in accordance with the manufacturer’s instruction. RNA extractions were treated with gDNA Eraser to avoid gDNA contamination. The mRNA abundance was measured with a FastStart Universal SYBR Green Master (ROX) (4913850001, Roche, Norwalk, CT, USA) in a 7500 Real-Time PCR System (Applied Biosystems Inc., Waltham, MA, USA). All melt curves of SYBR-based PCR targets were singly and discretely peaked. The reaction system contained 10 μL SYBR Green Master, 1 μL each primer, 1 μL cDNA, and 7 μL RNase Free dH_2_O. The primer sequences for each target gene and the reference gene are listed in Table S2. The reaction program consisted of an initial denaturation at 95 °C for 3 min, 40 cycles of denaturation at 95 °C for 15 s, annealing at 60 °C for 1 min, and extension at 60 °C for 1 min, with a final extension at 72 °C for 5 min. The relative expression of target genes was normalized to β-actin and calculated using the 2^−ΔΔCT^ method. The PCR reaction was repeated nine times for each treatment in vitro.

### 2.4. Measurement of Very Low-Density Lipoprotein Content

VLDL contents in the cell medium were measured by a Bovine VLDL ELISA Kit (H249, Nanjing Jiancheng Bioengineering Institute, Nanjing, China). The detection range of the kit was 0.05–20 μM; variation within a batch, coefficient of variation (CV) < 10%; variation between batches, CV < 12%.

### 2.5. Statistical Analysis

Data are presented as the mean ± standard error of the mean (SEM) and were subjected to statistical analysis with GraphPad Prism Version 5.0 (GraphPad InStat Software, San Diego, CA, USA). The qRT-PCR data were normally distributed and analyzed with *t*-test. Comparisons among groups in vitro were analyzed using the one-way ANOVA with Bonferroni correction. A *p*-value < 0.05 was statistically significant.

## 3. Results

### 3.1. Ad-SIRT3 Administration Promoted Very Low-Density LipoproteinSynthesis and Assembly in Cow Hepatocytes

**Figure 1:**
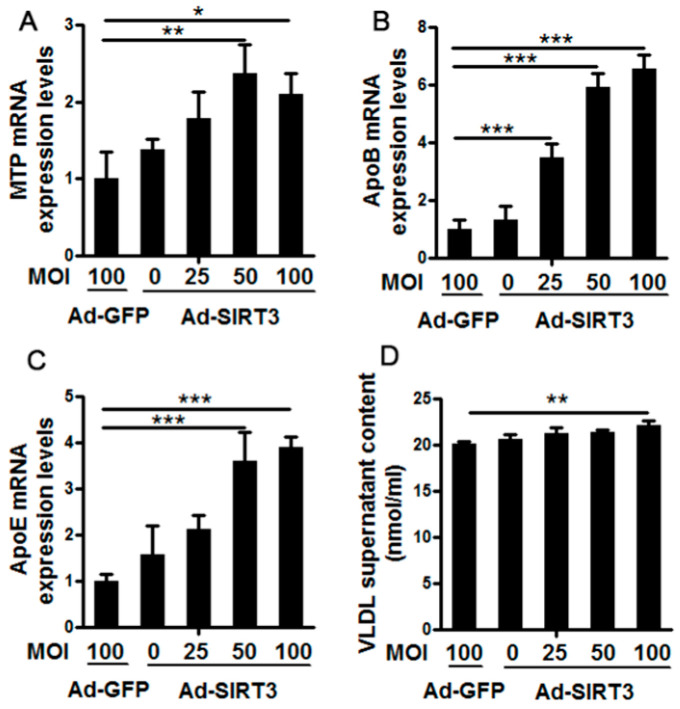
Effects of SIRT3 overexpression on the synthesis and secretion of Very Low-Density Lipoproteins (VLDL) in cow hepatocytes. Primary cow hepatocytes were cultured and treated with Ad-SIRT3 at 0, 25, 50, and 100 multiplicity of infection (MOI) for 6 h. Hepatocytes treated with Ad-GFP were used as the negative controls. After another 42 h of incubation with RPMI1640 medium, cells were harvested. The mRNA abundance of key proteins involved in the synthesis and assembly of VLDL, MTP (**A**), ApoB (**B**), and ApoE (**C**), were measured by qRT-PCR. The VLDL content in supernatants was measured by a commercial kit (**D**). For all bar plots shown, data are expressed as the mean ± SEM. * indicates *p* < 0.05; ** indicates *p* < 0.01; *** indicates *p* < 0.001 by the one-way ANOVA with Bonferroni correction. Results are representative of at least three independent measurements.

Results of SIRT3 overexpression on the synthesis and secretion of VLDL in cow hepatocytes are displayed in Figure 1. Compared with the Ad-GFP group, Ad-SIRT3 infection at MOI 50 and MOI 100 significantly increased the mRNA abundance of MTP (*p* < 0.05, Figure 1A). The mRNA abundance of ApoB100 displayed a dose-dependent manner upon Ad-SIRT3 infection with MOI 100 the highest (*p* < 0.001, Figure 1B). Similar to MTP mRNA abundance, Ad-SIRT3 treatment at MOI 50 and MOI 100 significantly increased mRNA abundance of ApoE compared with the Ad-GFP group (*p* < 0.001, Figure 1C). The VLDL content in the supernatants was significantly higher in the Ad-SIRT3 MOI 100 group compared with Ad-GFP (*p* < 0.01, Figure 1D).

### 3.2. SIRT3 Silencing Decreased Very Low-Density Lipoprotein Synthesis and Assembly in Cow Hepatocytes

**Figure 2:**
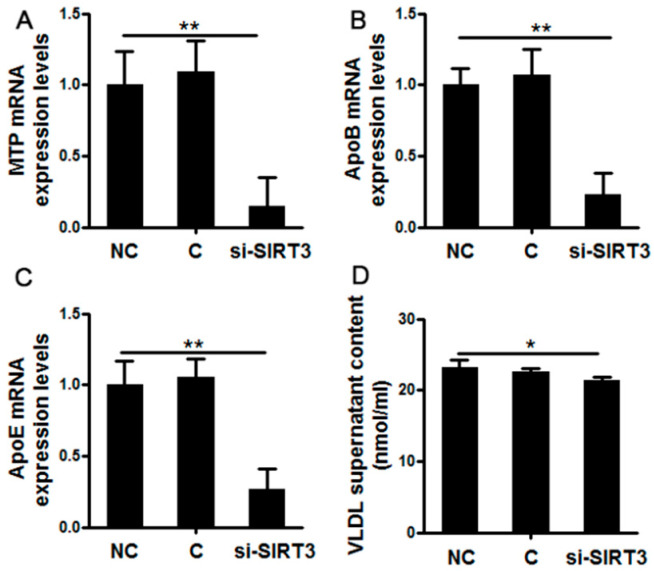
Effects of SIRT3 silencing on the synthesis and secretion of Very Low-Density Lipoprotein (VLDL) in cow hepatocytes. Primary cow hepatocytes were treated with si-SIRT3 by the lipofectamine 2000 regent for 6 h. Hepatocytes treated with si-RNA were used as the negative controls. After another 42 h of incubation with RPMI1640 medium, cells were harvested. The mRNA abundance of MTP (**A**), ApoB (**B**), and ApoE (**C**) were measured by qRT-PCR. The VLDL content in supernatants was measured by a commercial kit (**D**). NC, negative controls; C, blank controls; si-SIRT3, si-SIRT3 treatment group. For all bar plots shown, data are expressed as the mean ± SEM. * indicates *p* < 0.05; ** indicates *p* < 0.01 by the one-way ANOVA with Bonferroni correction. Results are representative of at least three independent measurements.

Figure 2 presents the effects of SIRT3 silencing on the synthesis and secretion of VLDL in cow hepatocytes. Compared with NC, si-SIRT3 administration significantly decreased mRNA abundance of MTP (*p* < 0.01, Figure 2A). The mRNA abundance of ApoB100 also decreased upon si-SIRT3 treatments (*p* < 0.01, Figure 2B). The si-SIRT3 treatment significantly reduced mRNA abundance of ApoE compared with the NC group (Figure 2C). The VLDL content in the supernatants was lower in the si-SIRT3 group compared with NC (*p* < 0.05, Figure 2D).

### 3.3. SIRT3 Reversed the Inhibition of Very Low-Density Lipoprotein Synthesis and Assembly by High Nonesterified Fatty Acids in Cow Hepatocytes

**Figure 3:**
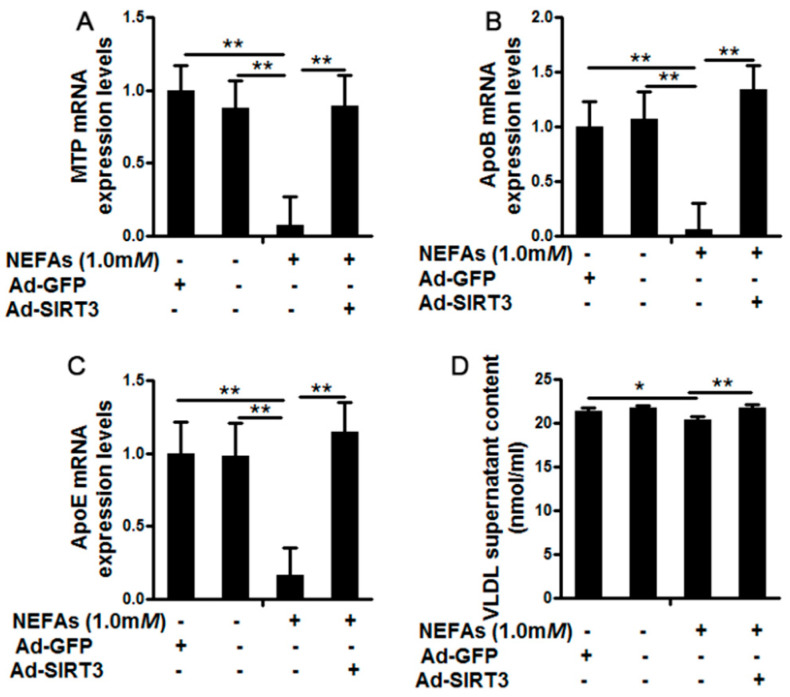
Effects of SIRT3 overexpression on the synthesis and secretion of Very Low-Density Lipoprotein (VLDL) in cow hepatocytes treated with nonesterified fatty acids (NEFA). Primary cow hepatocytes were treated with Ad-SIRT3 at 100 MOI for 30 h, serum starved for 6 h, and then treated with 1.0 m*M* NEFA for 12 h before cell harvest. Hepatocytes treated with Ad-GFP were used as the negative controls. The blank controls were hepatocytes incubated with RPMI1640 growth medium in parallel with the Ad-SIRT3 + NEFA group. The mRNA abundance of microsomal triglyceride transfer protein (MTP) (**A**), ApoB (**B**), and ApoE (**C**) were measured by qRT-PCR. The VLDL content in supernatants was measured by a commercial kit (**D**). For all bar plots shown, data are expressed as the mean ± SEM. * indicates *p* < 0.05; ** indicates *p* < 0.01 by the one-way ANOVA with Bonferroni correction. Results are representative of at least three independent measurements.

Figure 3 shows the effects of SIRT3 overexpression on the synthesis and secretion of VLDL in cow hepatocytes treated with NEFA. The mRNA abundances of the key proteins involved in VLDL synthesis, MTP (Figure 3A), ApoB100 (Figure 3B), and ApoE (Figure 3C), were lower in the 1 m*M* NEFA treatment group than in blank controls (*p* < 0.01). As a result, the VLDL content in supernatants was also lower in the NEFA group (Figure 3D). However, compared to the NEFA group, Ad-SIRT3 + 1 m*M* NEFA treatment significantly increased the mRNA abundances of MTP (Figure 3A), ApoB100 (Figure 3B), and ApoE (*p* < 0.01, Figure 3C). VLDL contents in the supernatants were higher in the NEFA +Ad-SIRT3 group than in the NEFA group (*p* < 0.01, Figure 3D).

## 4. Discussion

We found that SIRT3 overexpression significantly upregulated the mRNA expressions of MTP, ApoB100, and ApoE, and raised VLDL contents in the supernatants of cow hepatocytes. Conversely, SIRT3 silencing reversed those effects. In addition, compared with NEFA treatment alone, Ad-SIRT3 + NEFA significantly upregulated the mRNA abundance of MTP, ApoB100, and ApoE, and increased VLDL contents in the supernatants. Although much research is needed before targeting SIRT3 as a therapeutic agent for fatty liver treatment, this study provides in vitro evidence that SIRT3 may be effective in correcting fatty acid metabolism in cow hepatocytes.

Apolipoprotein B100 and ApoE are the most important apolipoproteins for VLDL synthesis [16]. Bovine ApoB100, MW at 534 kDa, is the largest apolipoprotein in VLDL, and is mainly synthesized in the liver of dairy cows. ApoB100 binds with TAG and stabilizes the nascent VLDL particles [16]. The nascent VLDL particles also acquire ApoE during the assembly process [17]. Both apolipoproteins have been shown to be intimately associated with fatty liver in dairy cows [18]. The hepatic mRNA abundance of ApoB100 is significantly reduced in cows with fatty liver [9,19]. Fatty liver cows also display lower serum concentrations of ApoB100/VLDL content [20,21,22]. Cows with fatty liver have a lower protein abundance of ApoB100 and ApoE in the liver [20]. These data suggest that restoring abundant ApoB100 and AopE may be helpful in preventing the progress of fatty liver. Our results indicated that SIRT3 overexpression increased ApoB100 expression in cow hepatocytes in a dose-dependent manner. A greater than six-fold increase of ApoB100 mRNA expression suggested SIRT3 is a robust stimulator for ApoB100. ApoE expression was also induced upon SIRT3 overexpression.

MTP is a transporter of newly synthesized TAG from outside of the endoplasmic reticulum to the lumen [23], where they were bound with apolipoproteins to form nascent VLDL. MTP plays a fundamental role in the assembly of VLDL [24]. We showed here MTP mRNA expression was regulated by SIRT3. As a result of increased expressions of ApoB100, ApoE, and MTP, the constituents for VLDL synthesis are more available. Therefore, VLDL in the supernatants are increased upon SIRT3 overexpression.

We then determined if SIRT3 silencing would inhibit the VLDL synthesis and assembly in cow hepatocytes. Administration of si-SIRT3 significantly suppressed the mRNA expression of MTP, ApoB100, and ApoE. VLDL secretion was lower in SIRT3-silencing hepatocytes. The results of SIRT3 overexpression/silencing assays indicate SIRT3 represents a potent regulator of VLDL in cow hepatocytes.

High blood NEFA concentration is closely associated with the development of fatty liver in dairy cows [25]. Specifically, high NEFA inhibit the synthesis and assembly of VLDL in cow hepatocytes [10]. The observation led us to investigate the role of SIRT3 in the synthesis and assembly in the event of high NEFA. Our data revealed that high NEFA inhibited mRNA expressions of MTP, ApoB100, and ApoE, and thus suppressed the secretion of VLDL into the supernatants. The result was in line with previous studies [10,20,26]. Those data together support a central role of high NEFA in the TAG accumulation in hepatocytes and further in the initiation and progress of fatty liver in cows, highlighting a feasibility of treating or preventing fatty liver by blocking the detrimental effects of excessive NEFA. There is growing evidence that SIRT3 is an antagonist to high NEFA. Ad-SIRT3 infection in response to 1 m*M* NEFA incubation significantly increased the mRNA abundance of MTP, ApoB100, and ApoE, and raised VLDL contents in the supernatants. This manifested that SIRT3 overexpression reversed the detrimental effects of high NEFA on VLDL synthesis and assembly. We previously reported that SIRT3 prevented TAG deposition in response to high NEFA in cow hepatocytes. The mechanism was shown to be via transcriptional regulation of key genes involved in fatty acid synthesis and oxidation. Together with the results in the present study, it is reasonable that SIRT3 serves as a major regulator of lipid homeostasis in cow hepatocytes encountering a high fatty acid influx, which always happens shortly after calving. In nonruminants, there have been some trials to mitigate nonalcoholic fatty liver diseases by targeting SIRT3 [27,28,29,30]. The possible mechanisms include activation of mitophagy, deacetylation of key enzymes in fatty acid metabolism, and improving mitochondrial respiratory capacity and redox homeostasis. However, there is a lack of in vivo evidence of SIRT3 on treatment of fatty liver in cows. Further investigations are needed to test the feasibility of SIRT3 on treatment of fatty liver cows, possibly by feeding SIRT3 agonist like resveratrol and honokiol to cows [31,32,33].

To conclude, high NEFA inhibited the synthesis and secretion of VLDL in cow hepatocytes; however, SIRT3 restored the synthesis and secretion of VLDL induced by high NEFA. The data provide a basis for further in vivo studies that test the possibility of SIRT3 in the treatment of fatty liver during the perinatal period.

## Data Availability

Data is contained within the article and Supplementary Material.

## Supplementary Materials

The following are available online at https://www.mdpi.com/article/10.3390/vetsci8070121/s1, Table S1: siRNA sequences for SIRT3 silencing, Table S2: The primers sequences for qRT-PCR.
