# Supplementary material for: Sirtuin 3 Restores Synthesis and Secretion of Very Low-Density Lipoproteins in Cow Hepatocytes Challenged with Nonesterified Fatty Acids In Vitro
Source: Vet Sci. 2021 Jun 30;8(7):121. doi: 10.3390/vetsci8070121 (PMC8310004; doi:10.3390/vetsci8070121)
Supplement: Supplementary file 1 [file vetsci-08-00121-s001.zip › vetsci-1217143-supplementary.pdf]

Table S1. siRNA sequences for SIRT3 silencing.

RNA	Sequence
si-SIRT3-1	sense: 5'-GGUGGAGGAUGGUCCAUAUTT-3'<br>antisense: 5'-AUAUGGACCAUCCUCCACCTT-3'
si-SIRT3-2	Sense: 5'-CCAGCGGCAUCCCAGACUUTT-3'<br>antisense: 5'-AAGUCUGGGAUGCCGCUGGTT-3'
si-SIRT3-3	sense: 5'-CCCUGACUCAAAGCUCGUUTT-3'<br>antisense: 5'-AACGAGCUUUGAGUCAGGGTT-3'
Negative Control	sense: 5'-UUCUCCGAACGUGUCACGUTT-3'<br>antisense: 5'-ACGUGACACGUUCGGAGAATT-3'

Table S2. The primers sequences for qRT-PCR.

Gene	Access number	Primer sequences (5'-3')	Product length (bp)
MTP	NM_001101834.1	F: CCATTCGGCATCTACTTACAGC<br>R: AGCCAGCATAGGAGTCAAGGTTCT	187
ApoB	BC_103438.1	F: GATACTCAGAACGGAGCAAT<br>R: GCACCAATCAGATAACAGGA	223
ApoE	NM_173991.2	F: TCCTGAATGACCTGGGTGTTG<br>R: TCTGTGGGTTGCCGTGGTG	217
β-actin	BC-142413.1	F: GCCCTGAGGCTCTCTTCCA<br>R: GCGGATGTCGACGTCACA	101
